# Genome Sequence of Campylobacter fetus subsp. *venerealis* NW_ED23, Isolated from Bovine Sheath Wash

**DOI:** 10.1128/MRA.00854-20

**Published:** 2020-10-01

**Authors:** Mpinda Edoaurd Tshipamba, Stephen Abiola Akinola, Lubanza Ngoma, Mulunda Mwanza

**Affiliations:** a Department of Animal Health, School of Agriculture, Faculty of Agriculture, Science, and Technology, Mafikeng Campus, North-West University, Mmabatho, South Africa; b Department of Microbiology, North West University, Mmabatho, South Africa; University of Southern California

## Abstract

Campylobacter fetus subsp. *venerealis* is the causative agent of bovine genital campylobacteriosis, which is mostly characterized by reproduction problems. The strain reported in this study was isolated from bull sheath wash in South Africa.

## ANNOUNCEMENT

Bovine genital campylobacteriosis belongs to list B of the notifiable diseases of the International Office of Epizootics (OIE) (1). A recent study reported that the Campylobacter fetus subsp. *venerealis* bv. intermedius genome sequence is available, but the same study confirmed that more analyses were needed to verify if there are extrachromosomal replicons (2). Furthermore, van der Graaf-van Bloois and coauthors revealed that an unassembled genome does not allow full identification of the core and accessory genome and the reconstruction of pathways and surface structures that might contribute to the pathogenicity of this biovar (3). An increase in the prevalence of this biovar has been noticed in South Africa (4). Hence, there was a need to sequence this particular strain to determine if there is any deviation from that previously reported.

The sample used for the isolation of Campylobacter fetus subsp. *venerealis* NW_ED23 was collected in the North-West Province, South Africa, specifically, in Mamusa District Municipality, cultured on tryptose blood agar base CM0233 (Oxoid, United Kingdom) mixed with 7% sheep blood, and supplemented with *Campylobacter* selective supplement (Skirrow) SR0068E (Oxoid). The plates were incubated at 37°C for 72 h in a 2.5-liter anaerobic jar (Oxoid) with a CampyGen CN0025A sachet (Oxoid) to create microaerophilic conditions (5). The genomic DNA (gDNA) was extracted from a single colony using a Zymo Research kit (Merck, South Africa). The PCR was conducted using MG3F and MG4R primers for the amplification of the 16S rRNA gene (6). The Basic Local Alignment Search Tool (BLAST) of the NCBI website was used to confirm identity and similarity (7), and the accession number obtained was MT138648. The gDNA from the pure colony based on Sanger identification confirmation was used for library preparation using a NEBNext Ultra II FS kit according to the manufacturer’s instructions and was used for the whole-genome sequencing.

The extracted gDNA sample was fragmented using an enzymatic approach (NEBNext Ultra II FS kit). The resulting DNA fragment was sized (200 to 500 bp) using AMPure XP beads. The fragment was end repaired, and Illumina-specific adapter sequences were ligated to each fragment. The sample was indexed, and a second size selection step was performed. The sample was then quantified using a fluorometric method and diluted to a standard concentration (4 nM). It was then sequenced on an Illumina NextSeq platform using a NEBNext Ultra II FS 300-cycle kit according to the manufacturer’s instructions to generate a total of 516,191,294 reads with 2 × 150-bp paired-end read length, and the coverage was 40×.

The sequence data were analyzed with the KBase platform with default parameters (8). The sequenced data were filtered for low-quality reads and adapter regions using Trimmomatic v0.36 (9) and FastQC v0.11.5. The genome assembly was performed using SPAdes v3.13.0 (10). Functional annotation of the whole-genome sequence was performed with the NCBI Prokaryotic Genome Annotation Pipeline (11) and Rapid Annotations using Subsystems Technology (RAST) v0.11 (12).

Campylobacter fetus subsp. *venerealis* NWU_ED23 has a total length of 1,880,748 bp assembled in 161 contigs with an average G+C content of 33.05%. The sequenced data generated contain 2,004 coding genes. The *N*_50_ value of the genome was 26,493 bp, while the *L*_50_ value was 22. The entire genome assembly contains 36 RNAs and 194 subsystems.

### Data availability.

The whole-genome shotgun project of Campylobacter fetus subsp. *venerealis* NWU_ED23 has been deposited in DDBJ/ENA/GenBank under the accession number JACASH000000000. The version published in this paper is JACASH010000000. The raw reads were also submitted to the NCBI SRA under accession number SRX8607292, BioSample number SAMN15356083, and BioProject number PRJNA641553.

## References

[1] McMillen L, Fordyce G, Doogan VJ, Lew AE 2006 Comparison of culture and a novel 5′ Taq nuclease assay for direct detection of Campylobacter fetus subsp. venerealis in clinical specimens from cattle. J Clin Microbiol 44:938–945. doi:10.1128/JCM.44.3.938-945.2006. 16517880 PMC1393111

[2] Iraola G, Pérez R, Naya H, Paolicchi F, Harris D, Lawley TD, Rego N, Hernández M, Calleros L, Carretto L, Velilla A, Morsella C, Méndez A, Gioffre A 2013 Complete genome sequence of Campylobacter fetus subsp. venerealis biovar intermedius, isolated from the prepuce of a bull. Genome Announc 1:e00526-13. doi:10.1128/genomeA.00526-13. 23908278 PMC3731832

[3] van der Graaf-van Bloois L, Miller WG, Yee E, Bono JL, Rijnsburger M, Campero C, Wagenaar JA, Duim B 2014 First closed genome sequence of Campylobacter fetus subsp. venerealis bv. intermedius. Genome Announc 2:e01246-13. doi:10.1128/genomeA.01246-13. PMC3916489 24503995

[4] Schmidt T, Venter EH, Picard J 2010 Evaluation of PCR assays for the detection of Campylobacter fetus in bovine preputial scrapings and the identification of subspecies in South African field isolates. J S Afr Vet Assoc 81:87–92. doi:10.4102/jsava.v81i2.111. 21247013

[5] Acke E, McGill K, Golden O, Jones B, Fanning S, Whyte P 2009 A comparison of different culture methods for the recovery of Campylobacter species from pets. Zoonoses Public Health 56:490–495. doi:10.1111/j.1863-2378.2008.01205.x. 19243565

[6] Schulze F, Bagon A, Müller W, Hotzel H 2006 Identification of Campylobacter fetus subspecies by phenotypic differentiation and PCR. J Clin Microbiol 44:2019–2024. doi:10.1128/JCM.02566-05. 16757592 PMC1489434

[7] Alayande KA, Aiyegoro OA, Nengwekhulu TM, Katata-Seru L, Ateba CN 2020 Integrated genome-based probiotic relevance and safety evaluation of Lactobacillus reuteri PNW1. PLoS One 15:e0235873. doi:10.1371/journal.pone.0235873. 32687505 PMC7371166

[8] Arkin AP, Cottingham RW, Henry CS, Harris NL, Stevens RL, Maslov S, Dehal P, Ware D, Perez F, Canon S, Sneddon MW, Henderson ML, Riehl WJ, Murphy-Olson D, Chan SY, Kamimura RT, Kumari S, Drake MM, Brettin TS, Glass EM, Chivian D, Gunter D, Weston DJ, Allen BH, Baumohl J, Best AA, Bowen B, Brenner SE, Bun CC, Chandonia J-M, Chia J-M, Colasanti R, Conrad N, Davis JJ, Davison BH, DeJongh M, Devoid S, Dietrich E, Dubchak I, Edirisinghe JN, Fang G, Faria JP, Frybarger PM, Gerlach W, Gerstein M, Greiner A, Gurtowski J, Haun HL, He F, Jain R, 2018 KBase: the United States Department of Energy Systems Biology Knowledgebase. Nat Biotechnol 36:566–569. doi:10.1038/nbt.4163. 29979655 PMC6870991

[9] Bolger AM, Lohse M, Usadel B 2014 Trimmomatic: a flexible trimmer for Illumina sequence data. Bioinformatics 30:2114–2120. doi:10.1093/bioinformatics/btu170. 24695404 PMC4103590

[10] Bankevich A, Nurk S, Antipov D, Gurevich AA, Dvorkin M, Kulikov AS, Lesin VM, Nikolenko SI, Pham S, Prjibelski AD, Pyshkin AV, Sirotkin AV, Vyahhi N, Tesler G, Alekseyev MA, Pevzner PA 2012 SPAdes: a new genome assembly algorithm and its applications to single-cell sequencing. J Comput Biol 19:455–477. doi:10.1089/cmb.2012.0021. 22506599 PMC3342519

[11] Haft DH, DiCuccio M, Badretdin A, Brover V, Chetvernin V, O’Neill K, Li W, Chitsaz F, Derbyshire MK, Gonzales NR, Gwadz M, Lu F, Marchler GH, Song JS, Thanki N, Yamashita RA, Zheng C, Thibaud-Nissen F, Geer LY, Marchler-Bauer A, Pruitt KD 2018 RefSeq: an update on prokaryotic genome annotation and curation. Nucleic Acids Res 46:D851–D60. doi:10.1093/nar/gkx1068. 29112715 PMC5753331

[12] Aziz RK, Bartels D, Best AA, DeJongh M, Disz T, Edwards RA, Formsma K, Gerdes S, Glass EM, Kubal M, Meyer F, Olsen GJ, Olson R, Osterman AL, Overbeek RA, McNeil LK, Paarmann D, Paczian T, Parrello B, Pusch GD, Reich C, Stevens R, Vassieva O, Vonstein V, Wilke A, Zagnitko O 2008 The RAST server: Rapid Annotations using Subsystems Technology. BMC Genomics 9:75. doi:10.1186/1471-2164-9-75. 18261238 PMC2265698

